# Artificial intelligence–based alopecia assessment: A proof of concept for enhancing accuracy and objectivity in hair loss measurement

**DOI:** 10.1016/j.jdcr.2025.09.023

**Published:** 2025-10-09

**Authors:** Evan Chan, Kaitlyn Ramsay, Rachel Tyli, Ryan S.Q. Geng, Tahirih Nasseri, Vincent Piguet, Robert D.J. Fraser, Sheila C. Wang

**Affiliations:** a Massey College, University of Toronto, Toronto, Ontario, Canada; b Department of Medicine, Temerty Faculty of Medicine, University of Toronto, Toronto, Ontario, Canada; c Department of Laboratory Medicine and Pathobiology, Temerty Faculty of Medicine, University of Toronto, Toronto, Ontario, Canada; d Swift Medical, Toronto, Ontario, Canada; e Division of Dermatology, Department of Medicine, Women’s College Hospital, Toronto, Ontario, Canada; f Arthur Labatt Family School of Nursing, Faculty of Health Sciences, Western University, London, Ontario, Canada

**Keywords:** alopecia areata, artificial intelligence, computer vision, SALT scores

## Introduction

Alopecia areata (AA) is characterized by nonscarring hair loss, with significant psychosocial impact.^1^^,^^2^ Accurate hair loss assessment is important for monitoring disease progression, evaluating treatment efficacy, and guiding therapeutic decisions. Traditional methods rely on visual estimation and manual measurements, which are time-consuming and imprecise. The Severity of Alopecia Tool (SALT) is the standardized method for quantifying hair loss in AA.^3^, ^4^, ^5^ Trichoscopy allows for detailed visualization of subtle hair and scalp features, aiding in the differentiation of AA from other hair disorders and in monitoring treatment response by identifying early signs of hair regrowth. This technique requires specialized equipment and examiner expertise which limits availability in clinical settings.^6^

Recent advancements in artificial intelligence (AI) and computer vision offer promising solutions to these challenges. Previous studies have demonstrated that AI-based systems can automate the segmentation and measurement of hair loss, reducing human error and subjectivity.^7^^,^^8^ Deep learning models have been employed to assess hair loss severity and assess early follicular hair regrowth when monitoring treatment responses objectively.^9^ The AI-imaging system (Skin and Wound–Swift Medical) leverages millions of calibrated images for segmentation of AI algorithms. The advantage of this tool over other existing systems is that the software is accessible by smartphone and can be used at the bedside. It calculates an accurate surface area measurement of AA, a critical component to SALT scores, using advanced imaging technology and edge detection algorithms. This case demonstrates a proof of concept for tracking and managing AA using an AI-based assessment tool. For this case of AA, AI is used to calculate accurate areas of alopecia and trichoscopy was used to detect of early follicular regrowth, providing a faster, more comprehensive and objective evaluation of disease progression and treatment response.

## Patient case report

**Fig 1:**
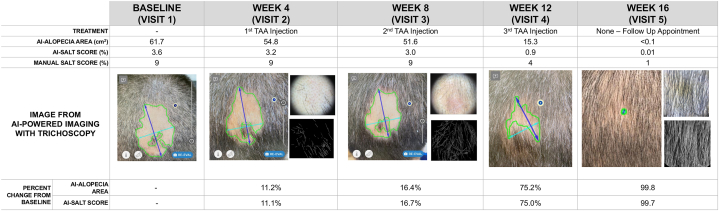
Changes in AI-SALT scores, manual-SALT scores, and alopecia area over 16 weeks with 10 mg/cc intralesional triamcinolone acetonide treatments. Baseline (visit 1), week 4, week 8, week 12, and week 16 (last follow-up). Trichoscopy with and without edge detection is shown for week 4, week 8, and week 16. *SALT*, Severity of Alopecia Tool.

A 47-year-old male patient with no AA-associated comorbidities (antinuclear antibody negative, thyroid-stimulating hormone normal, glucose normal, and complete blood count normal), no known allergies, and not currently on any medications was presented to clinic for evaluation of scalp hair loss progressing for more than 1 year. Examination revealed a solitary well-circumscribed nonerythematous alopecia area on the mid-occiput. Trichoscopy with manual edge detection showed tapering/broken hairs, exclamation mark hairs, yellow and black dots, and short vellus hairs, consistent with AA (Fig 1). The patient was seen at 4-week intervals for a total of 16 weeks and treated with 10 mg/cc of intralesional triamcinolone acetonide at each visit. The alopecia was imaged using an AI tool that provided automated area measurements (AI-Area) to calculate SALT scores (AI-SALT score) and percent change of alopecia area from baseline (Fig 1). Manual SALT scores were recorded. At each visit, the percentage change from baseline for AI-SALT score and alopecia area was calculated.

A notable aspect of disease progression at week 16 is that the figure appears to show ongoing alopecia <1% (Fig 1), but the area is filled with white regrown hairs (seen on trichoscopy). This explains the 99% improvement in SALT score despite visual appearance of minimal persistent alopecia (white hairs were visible starting in the week 8 trichoscopy). At final visit, based on trichoscopy, a hair density of 162 hairs/cm^2^ was calculated. Normal hair density for a healthy adult ranges from 100 to 150 hairs/cm^2^.

## Discussion

In clinical trials, SALT scores are a standardized method for assessing AA.^3^^,^^4^ SALT scores are rarely used in day-to-day practice because they are time-consuming in nature. They have other limitations including high inter-rater variability and inability to account for diverse head shapes. Instead, clinicians often rely on subjective visual assessments and images to determine if alopecia is improving.

Using the AI tool to assess this patient’s alopecia, demonstrates fast, easy-to-use AI-powered imaging via smartphone to precisely and objectively quantify alopecia for clinical evaluation. Furthermore, percentage change of alopecia area provides more accurate and sensitive assessment than the manual SALT system demonstrated by the AI-SALT scores’s finer decimal-level precision. This tool’s sensitivity captured incremental progress between visits 1 to 3 (Fig 1), which manual SALT assessments did not detect. Demonstrating incremental improvements was crucial, as quantitative documentation of change was essential for enhancing patient engagement and motivation. Notably, at week 8, the patient considered discontinuing treatment due to a lack of noticeable improvements. However, the AI tool’s ability to demonstrate clear progress, combined with trichoscopic images showing hair regrowth, motivated him to continue. This highlights the importance of detecting and quantifying subtle improvements to maintain patient motivation and achieve successful treatment outcomes.

Monitoring and documenting the extent of alopecia is crucial in clinical settings for assessing treatment efficacy. Insurers typically require precise evidence of disease severity, including accurate SALT scores, to approve treatment coverage. The global incidence of AA increased by 49% from 1990 to 2019,^10^ fueling the demand for accurate measurement tools as new treatments are being developed in clinical trials. Today, AI tools are increasingly being used to assess skin disorders, and we are beginning to integrate these tools into our daily clinical practice. In this case, the integration of AI enabled more accurate monitoring and patient engagement, suggesting potential to improve care through personalized and informed management of AA.

However, this tool has limitations, particularly in cases of androgenetic alopecia, where diffuse hair thinning lacks distinct patches. Combining trichoscopy with AI to quantify individual hairs or follicular units enhances assessment, as demonstrated here. The tool’s performance should be evaluated in more severe or widespread alopecia to validate utility beyond single-patch presentations. Additionally, validation across a range of hair textures, colors, and patterns of hair loss is needed to support its use in diverse patient populations.

Here, we present a proof-of-concept for integrating AI tools in clinical settings to assess and monitor AA, demonstrating the potential to deliver precise and objective data that inform treatment decisions and patient engagement. However, this is a proof of concept used on 1 patient, and further validation is necessary to ensure broader applicability.

## Conflicts of interest

R.D.J.F. is employed by Swift Medical as GM & VP, Advanced Clinical Solutions. S.W. is a co-founder and Physician Scientist at Swift Medical. V.P. has received grants from AbbVie, Bausch Health, Boehringer Ingelheim, Bristol Myers Squibb, Celgene, Eli Lilly, Incyte, Janssen, LEO Pharma, L'Oréal, Novartis, Organon, Pfizer, Sandoz, and Sanofi; received payment or honoraria for speaking engagement from Sanofi; participated on an advisory board for LEO Pharma, Novartis, Sanofi, Union Therapeutics, Abbvie, and UCB; and received equipment donation from L'Oréal. Authors E.C., K.R., R.T., and R.S.Q.G. have no conflicts to declare.

## Supplementary data

Authorship Explanation Combined
