# Supplementary material for: Artificial intelligence–based alopecia assessment: A proof of concept for enhancing accuracy and objectivity in hair loss measurement
Source: JAAD Case Rep. 2025 Oct 9;66:131–3. doi: 10.1016/j.jdcr.2025.09.023 (PMC12805230; doi:10.1016/j.jdcr.2025.09.023)
Supplement: Authorship Explanation Combined [file mmc1.pdf]

We have added Tahirih Nasseri to the authorship line of the manuscript titled *"AI-Based Alopecia Assessment: A Proof of Concept for Enhancing Accuracy and Objectivity in Hair Loss Measurement"* based on their substantial contributions during the revision process.

- **Tahirih Nasseri** was added as an author in recognition of her extensive contributions to editing and refining the revised manuscript.

Ms Nasseri meets the authorship criteria and have provided their written consent to be added. All contributing authors have been notified of the updated authorship order and are in the process of providing their formal consent as per journal requirements.

Subject: Re: Submission to JAAD Case Reports requires action
Date: Wednesday, July 30, 2025 at 11:48:56 Eastern Daylight Saving Time
From: Evan Chan
To: Rachel Tyli

I **Evan Chan** consent to **Tahirih Nasseri** to the authorship line of JAAD manuscript, "AI-Based Alopecia Assessment: A Proof of Concept for Enhancing Accuracy and Objectivity in Hair Loss Measurement. I consent to the following author order: Evan Chan, BM1*, Kaitlyn Ramsay, PhD2*, Rachel Tyli, BASc3*, Ryan S.Q. Geng, MSc2, Tahirih Nasseri, BSc 2, Vincent Piguet MD, PhD, FRCP 5, Robert D.J. Fraser, BScN, MN, RN4,5, Sheila C. Wang, MD, PhD, FRCPC2,4,6, *all authors contributed equally"

EVAN CHAN
PhD Student
Governor, Governing Council
Junior Fellow, Massey College
Research Fellow, Swift Medical

University of Toronto, Massey College
evankp.chan@mail.utoronto.ca
4 Devonshire Pl. | Toronto, ON M5S 2E1

From: Rachel Tyli <rachel.tyli@utoronto.ca>
Sent: Wednesday, July 30, 2025 11:18 AM
To: Evan Chan <evankp.chan@mail.utoronto.ca>
Subject: FW: Submission to JAAD Case Reports requires action

Hi Evan,

I received the following about our AA paper resubmission (I've attached the manuscript to the email for you to see the authorship order along with the table of changes). (see below in red).

> Please upload an MS Word or PDF file explaining the reason for the change and showing screenshots of each author's emailed consent to changing the authorship line, with the author(s) being added and the order of authors explicitly stated in each author's email. The author(s) who is being added must also consent to the change. The format of the email from each author could be as follows:
> "I [ADD Author First/Last Names] consent to [Adding] [Author First and Last Name] to the authorship line of JAAD manuscript, "[Add Manuscript Title]. I consent to the following author order: [Add all first/last names of authors in the correct order]"

We added Tahirih because she did a lot of editing for the manuscript revision and Sheila advised I add myself as third author as I made the figure, wrote and edited most of the manuscript.

I was wondering if you could send me back a statement with the following:
“I [**ADD Author First/Last Names**] consent to [Adding] [Tahirih Nasseri] to the authorship line of JAAD manuscript, “[AI-Based Alopecia Assessment: A Proof of Concept for Enhancing Accuracy and Objectivity in Hair Loss Measurement]. I consent to the following author order: [Evan Chan, BM1*, Kaitlyn Ramsay, PhD2*, Rachel Tyli, BASc3*, Ryan S.Q. Geng, MSc2, Tahirih Nasseri, BSc 2, Vincent Piguet MD, PhD, FRCP 5, Robert D.J. Fraser, BScN, MN, RN4,5, Sheila C. Wang, MD, PhD, FRCPC2,4,6,] *all authors contributed equally”

If you could send this email to Kaitlyn, that would be great. I’m trying to track everyone else down on my end.

Best,
Rachel

From: em.jdcr.0.95153d.2859d252@editorialmanager.com <em.jdcr.0.95153d.2859d252@editorialmanager.com> on behalf of JDCR <em@editorialmanager.com>
Date: Wednesday, July 30, 2025 at 07:51
To: Rachel Tyli <rachel.tyli@utoronto.ca>
Subject: Submission to JAAD Case Reports requires action

Journal: JAAD Case Reports
Title: AI-Based Alopecia Assessment: A Proof of Concept for Enhancing Accuracy and Objectivity in Hair Loss Measurement

Dear Ms Rachel Tyli,

We have received the above referenced manuscript you submitted to JAAD Case Reports. However, further action is required to complete the submission.

Technical comments (if applicable):

1. A change in authorship has been noted between this version of your manuscript and the original. Per the JAAD Guide for Authors (https://www.jaad.org/content/authorinfo), “Once a manuscript has been submitted, the order of authorship (including adding or removing authors) cannot be changed without a request signed by all authors and approved by the Editor."

Please upload an MS Word or PDF file explaining the reason for the change and showing screenshots of each author's emailed consent to changing the authorship line, with the author(s) being added and the order of authors explicitly stated in each author's email. The author(s) who is being added must also consent to the change. The format of the email from each author could be as follows:
“I [ADD Author First/Last Names] consent to [Adding] [Author First and Last Name] to the authorship line of JAAD manuscript, “[Add Manuscript Title]. I consent to the following author order:

[Add all first/last names of authors in the correct order]”

2. The image resolution for Figure 1 is too low. The minimum resolution for a figure needs to be 300dpi.

3. The corresponding author on the title page does not match the corresponding author in Editorial Manager. Please ensure that the corresponding author matches in both places.

When you are ready to proceed with your submission, please log in as an author at https://www.editorialmanager.com/jdcr/, and navigate to the "Submissions Sent Back to Author" folder. There you can edit your submission by clicking "Edit submission" under the "Action Link" menu.

Thank you for considering this journal, and we look forward to receiving your submission.

Kind regards,

JAAD Case Reports

1. A change in authorship has been noted between this version of your manuscript and the original. Per the JAAD Guide for Authors (https://www.jaad.org/content/authorinfo), “Once a manuscript has been submitted, the order of authorship (including adding or removing authors) cannot be changed without a request signed by all authors and approved by the Editor."

Please upload an MS Word or PDF file explaining the reason for the change and showing screenshots of each author's emailed consent to changing the authorship line, with the author(s) being added and the order of authors explicitly stated in each author's email. The author(s) who is being added must also consent to the change. The format of the email from each author could be as follows:
“I [ADD Author First/Last Names] consent to [Adding] [Author First and Last Name] to the authorship line of JAAD manuscript, “[Add Manuscript Title]. I consent to the following author order: [Add all first/last names of authors in the correct order]”

2. The image resolution for Figure 1 is too low. The minimum resolution for a figure needs to be 300dpi.

3. The corresponding author on the title page does not match the corresponding author in Editorial Manager. Please ensure that the corresponding author matches in both places.

More information and support

%CUSTOM_AUTHORSUPPORT%

FAQ: How can I reset a forgotten password?

https://service.elsevier.com/app/answers/detail/a_id/28452/supporthub/publishing/

For further assistance, please visit our customer service site: https://service.elsevier.com/app/home/supporthub/publishing/. Here you can search for solutions on a range of topics, find answers to frequently asked questions, and learn more about Editorial Manager via interactive tutorials. You can also talk 24/7 to our customer support team by phone and 24/7 by live chat and email

#AU_JDCR#

To ensure this email reaches the intended recipient, please do not delete the above code

---

In compliance with data protection regulations, you may request that we remove your personal registration details at any time. (Use the following URL: https://www.editorialmanager.com/jdcr/login.asp?a=r). Please contact the publication office if you have any questions.

Subject: F/U
Date: Friday, August 1, 2025 at 14:59:36 Eastern Daylight Saving Time
From: ramsayk@student.ubc.ca
To: Rachel Tyli

You don't often get email from ramsayk@student.ubc.ca. Learn why this is important

Hey Rachel!

I, Kaitlyn Ramsay consent to [Adding] [Tahirih Nasseri] to the authorship line of JAAD manuscript, "[AI-Based Alopecia Assessment: A Proof of Concept for Enhancing Accuracy and Objectivity in Hair Loss Measurement]. I consent to the following author order: [Evan Chan, BM1*, Kaitlyn Ramsay, PhD2*, Rachel Tyli, BASc3*, Ryan S.Q. Geng, MSc2, Tahirih Nasseri, BSc 2, Vincent Piguet MD, PhD, FRCP 5, Robert D.J. Fraser, BScN, MN, RN4,5, Sheila C. Wang, MD, PhD, FRCPC2,4,6,] *all authors contributed equally"

Envoyé à partir de Outlook pour Android

Subject: Authorship JAAD Article
Date: Sunday, August 3, 2025 at 20:23:28 Eastern Daylight Saving Time
From: Rachel Tyli
To: Rachel Tyli

"I Rachel Tyli consent to adding Tahirih Nasseri to the authorship line of JAAD manuscript, "[AI-Based Alopecia Assessment: A Proof of Concept for Enhancing Accuracy and Objectivity in Hair Loss Measurement]. I consent to the following author order: [Evan Chan, BM1*, Kaitlyn Ramsay, PhD2*, Rachel Tyli, BASc3*, Ryan S.Q. Geng, MSc2, Tahirih Nasseri, BSc 2, Vincent Piguet MD, PhD, FRCP 5, Robert D.J. Fraser, BScN, MN, RN4,5, Sheila C. Wang, MD, PhD, FRCPC2,4,6,] *all authors contributed equally"

Subject: Re: Authorship for AA Case Report Submission
Date: Wednesday, July 30, 2025 at 16:36:54 Eastern Daylight Saving Time
From: Ryan Geng
To: Rachel Tyli

Hey Rachel,

Here is the statement:

"I [**Ryan Geng**] consent to [Adding] [Tahirih Nasseri] to the authorship line of JAAD manuscript, "[AI-Based Alopecia Assessment: A Proof of Concept for Enhancing Accuracy and Objectivity in Hair Loss Measurement]. I consent to the following author order: [Evan Chan, BM1*, Kaitlyn Ramsay, PhD2*, Rachel Tyli, BASc3*, Ryan S.Q. Geng, MSc2, Tahirih Nasseri, BSc 2, Vincent Piguet MD, PhD, FRCP 5, Robert D.J. Fraser, BScN, MN, RN4,5, Sheila C. Wang, MD, PhD, FRCPC2,4,6,] *all authors contributed equally"

Regards,

Ryan

From: Rachel Tyli <rachel.tyli@utoronto.ca>
Sent: 30 July 2025 1:30 PM
To: Rob Fraser <rob.fraser@swiftmedical.com>; Sheila Wang <sheila.wang@utoronto.ca>; QH Wang <wang.sqh@gmail.com>; Ryan Geng <ryan.geng@mail.utoronto.ca>
Cc: Tahirih Nasseri <tahirih.nasseri@mail.utoronto.ca>
Subject: Authorship for AA Case Report Submission

Hi Team,

I received the following about our AA paper resubmission (I've attached the manuscript to the email for you to see the authorship order along with the table of changes). (see below in red).

> Please upload an MS Word or PDF file explaining the reason for the change and showing screenshots of each author's emailed consent to changing the authorship line, with the author(s) being added and the order of authors explicitly stated in each author's email. The author(s) who is being added must also consent to the change. The format of the email from each author could be as follows:
> "I [ADD Author First/Last Names] consent to [Adding] [Author First and Last Name] to the authorship line of JAAD manuscript, "[Add Manuscript Title]. I consent to the following author order: [Add all first/last names of authors in the correct order]"

We added Tahirih because she did a lot of editing for the manuscript revision and Sheila advised I add myself as third author as I made the figure, wrote and edited most of the manuscript.

I was wondering if you could send me back a statement with the following:
“I [**ADD Author First/Last Names**] consent to [Adding] [Tahirih Nasseri] to the authorship line of JAAD manuscript, “[AI-Based Alopecia Assessment: A Proof of Concept for Enhancing Accuracy and Objectivity in Hair Loss Measurement]. I consent to the following author order: [Evan Chan, BM1*, Kaitlyn Ramsay, PhD2*, Rachel Tyli, BASc3*, Ryan S.Q. Geng, MSc2, Tahirih Nasseri, BSc 2, Vincent Piguet MD, PhD, FRCP 5, Robert D.J. Fraser, BScN, MN, RN4,5, Sheila C. Wang, MD, PhD, FRCPC2,4,6,] *all authors contributed equally”

Sheila, if you could send this email to Vincent, that would be great. I’m trying to track everyone else down on my end. So far, Tahirih and Evan have sent me back their emails.

Best,
Rachel

Subject: Re: Submission to JAAD Case Reports requires action
Date: Wednesday, July 30, 2025 at 13:04:53 Eastern Daylight Saving Time
From: Tahirih Nasseri
To: Rachel Tyli

Hi Rachel,

I **Tahirih Nasseri** consent to be added to the authorship line of JAAD manuscript, "[AI-Based Alopecia Assessment: A Proof of Concept for Enhancing Accuracy and Objectivity in Hair Loss Measurement]. I consent to the following author order: [Evan Chan, BM1*, Kaitlyn Ramsay, PhD2*, Rachel Tyli, BASc3*, Ryan S.Q. Geng, MSc2, Tahirih Nasseri, BSc 2, Vincent Piguet MD, PhD, FRCP 5, Robert D.J. Fraser, BScN, MN, RN4,5, Sheila C. Wang, MD, PhD, FRCPC2,4,6,] *all authors contributed equally

Thank you!
Tahirih

Tahirih Nasseri *(she/her)*
MD Student, Class of 2027
Temerty Faculty of Medicine, University of Toronto

From: Rachel Tyli <rachel.tyli@utoronto.ca>
Sent: July 30, 2025 11:27 AM
To: Tahirih Nasseri <tahirih.nasseri@mail.utoronto.ca>
Subject: FW: Submission to JAAD Case Reports requires action

Does this sound okay?

Hi Team,

I received the following about our AA paper resubmission (I've attached the manuscript to the email for you to see the authorship order along with the table of changes). (see below in red).

> Please upload an MS Word or PDF file explaining the reason for the change and showing screenshots of each author's emailed consent to changing the authorship line, with the author(s) being added and the order of authors explicitly stated in each author's email. The author(s) who is being added must also consent to the change. The format of the email from each author could be as follows:
> "I [ADD Author First/Last Names] consent to [Adding] [Author First and Last Name] to the authorship line of JAAD manuscript, "[Add Manuscript Title]. I consent to the following author order: [Add all first/last names of authors in the correct order]"

We added Tahirih because she did a lot of editing for the manuscript revision and Sheila advised I add

myself as third author as I made the figure, wrote and edited most of the manuscript.

I was wondering if you could send me back a statement with the following:
"I [**ADD Author First/Last Names**] consent to [Adding] [Tahirih Nasseri] to the authorship line of JAAD manuscript, "[AI-Based Alopecia Assessment: A Proof of Concept for Enhancing Accuracy and Objectivity in Hair Loss Measurement]. I consent to the following author order: [Evan Chan, BM1*, Kaitlyn Ramsay, PhD2*, Rachel Tyli, BASc3*, Ryan S.Q. Geng, MSc2, Tahirih Nasseri, BSc 2, Vincent Piguet MD, PhD, FRCP 5, Robert D.J. Fraser, BScN, MN, RN4,5, Sheila C. Wang, MD, PhD, FRCPC2,4,6,] *all authors contributed equally"

Please let me know if you have any other questions for me. I would like to get everything in by the end of this week as that is our deadline for re-submission.

Best,
Rachel

From: em.jdcr.0.95153d.2859d252@editorialmanager.com <em.jdcr.0.95153d.2859d252@editorialmanager.com> on behalf of JDCR <em@editorialmanager.com>
Date: Wednesday, July 30, 2025 at 07:51
To: Rachel Tyli <rachel.tyli@utoronto.ca>
Subject: Submission to JAAD Case Reports requires action

Journal: JAAD Case Reports
Title: AI-Based Alopecia Assessment: A Proof of Concept for Enhancing Accuracy and Objectivity in Hair Loss Measurement

Dear Ms Rachel Tyli,

We have received the above referenced manuscript you submitted to JAAD Case Reports. However, further action is required to complete the submission.

Technical comments (if applicable):

1. A change in authorship has been noted between this version of your manuscript and the original. Per the JAAD Guide for Authors (https://www.jaad.org/content/authorinfo), "Once a manuscript has been submitted, the order of authorship (including adding or removing authors) cannot be changed without a request signed by all authors and approved by the Editor."

Please upload an MS Word or PDF file explaining the reason for the change and showing screenshots of each author's emailed consent to changing the authorship line, with the author(s) being added and the order of authors explicitly stated in each author's email. The author(s) who is being added must also consent to the change. The format of the email from each author could be as follows:
"I [ADD Author First/Last Names] consent to [Adding] [Author First and Last Name] to the authorship line of JAAD manuscript, "[Add Manuscript Title]. I consent to the following author order: [Add all first/last names of authors in the correct order]"

2. The image resolution for Figure 1 is too low. The minimum resolution for a figure needs to be 300dpi.

3. The corresponding author on the title page does not match the corresponding author in Editorial Manager. Please ensure that the corresponding author matches in both places.

When you are ready to proceed with your submission, please log in as an author at https://www.editorialmanager.com/jdcr/, and navigate to the "Submissions Sent Back to Author" folder. There you can edit your submission by clicking "Edit submission" under the "Action Link" menu.

Thank you for considering this journal, and we look forward to receiving your submission.

Kind regards,

JAAD Case Reports

1. A change in authorship has been noted between this version of your manuscript and the original. Per the JAAD Guide for Authors (https://www.jaad.org/content/authorinfo), “Once a manuscript has been submitted, the order of authorship (including adding or removing authors) cannot be changed without a request signed by all authors and approved by the Editor."

Please upload an MS Word or PDF file explaining the reason for the change and showing screenshots of each author's emailed consent to changing the authorship line, with the author(s) being added and the order of authors explicitly stated in each author's email. The author(s) who is being added must also consent to the change. The format of the email from each author could be as follows:
“I [ADD Author First/Last Names] consent to [Adding] [Author First and Last Name] to the authorship line of JAAD manuscript, “[Add Manuscript Title]. I consent to the following author order: [Add all first/last names of authors in the correct order]”

2. The image resolution for Figure 1 is too low. The minimum resolution for a figure needs to be 300dpi.

3. The corresponding author on the title page does not match the corresponding author in Editorial Manager. Please ensure that the corresponding author matches in both places.

More information and support

%CUSTOM_AUTHORSUPPORT%

FAQ: How can I reset a forgotten password?

https://service.elsevier.com/app/answers/detail/a_id/28452/supporthub/publishing/

For further assistance, please visit our customer service site: https://service.elsevier.com/app/home/supporthub/publishing/. Here you can search for solutions on a range of topics, find answers to frequently asked questions, and learn more about Editorial Manager via interactive tutorials. You can also talk 24/7 to our customer support team by phone and 24/7 by live chat and email

#AU_JDCR#

To ensure this email reaches the intended recipient, please do not delete the above code

---

In compliance with data protection regulations, you may request that we remove your personal registration details at any time. (Use the following URL: https://www.editorialmanager.com/jdcr/login.asp?a=r). Please contact the publication office if you have any questions.

Subject: Re: Authorship for AA Case Report Submission
Date: Monday, August 4, 2025 at 14:27:07 Eastern Daylight Saving Time
From: Rob Fraser
To: Rachel Tyli
CC: Sheila Wang

Hi Rachel,

I Robert Fraser consent to [Adding] [Tahirih Nasseri] to the authorship line of JAAD manuscript, "[AI-Based Alopecia Assessment: A Proof of Concept for Enhancing Accuracy and Objectivity in Hair Loss Measurement]. I consent to the following author order: [Evan Chan, BM1*, Kaitlyn Ramsay, PhD2*, Rachel Tyli, BASc3*, Ryan S.Q. Geng, MSc2, Tahirih Nasseri, BSc 2, Vincent Piguet MD, PhD, FRCP 5, Robert D.J. Fraser, BScN, MN, RN4,5, Sheila C. Wang, MD, PhD, FRCPC2,4,6,] *all authors contributed equally

Please let me know if you need anything else.

ROB FRASER MN RN NSWOC WOCC(C)
GM & VP, Advanced Clinical Solutions
Pronouns: He/Him
416.881.9634
(Toronto Based | Time Zone: EST)
OOO: July 28th-Aug 4th
Swift is proud to be recognized in Newsweek's Best Digital Health Companies in 2024.

From: Rachel Tyli <rachel.tyli@utoronto.ca>
Sent: August 3, 2025 8:25 PM
To: Rob Fraser <rob.fraser@swiftmedical.com>
Cc: Sheila Wang <sheila.wang@utoronto.ca>
Subject: Re: Authorship for AA Case Report Submission

CAUTION: External Sender. Do not click links or open attachments unless you recognize the sender and know the content is safe.

Hi Rob,

Just wondering if I could get the following statement from you to add Tahirih to the authorship for the AA Article. I'm just waiting on you for your email.

"I [**ADD Author First/Last Names**] consent to [Adding] [Tahirih Nasseri] to the authorship line of JAAD manuscript, "[AI-Based Alopecia Assessment: A Proof of Concept for Enhancing Accuracy

and Objectivity in Hair Loss Measurement]. I consent to the following author order: [Evan Chan, BM1*, Kaitlyn Ramsay, PhD2*, Rachel Tyli, BASc3*, Ryan S.Q. Geng, MSc2, Tahirih Nasseri, BSc 2, Vincent Piguet MD, PhD, FRCP 5, Robert D.J. Fraser, BScN, MN, RN4,5, Sheila C. Wang, MD, PhD, FRCPC2,4,6,] *all authors contributed equally"

Best,
Rachel

From: Rachel Tyli <rachel.tyli@utoronto.ca>
Date: Wednesday, July 30, 2025 at 13:30
To: Rob Fraser <rob.fraser@swiftmedical.com>, Sheila Wang <sheila.wang@utoronto.ca>, QH Wang <wang.sqh@gmail.com>, Ryan Geng <ryan.geng@mail.utoronto.ca>
Cc: Tahirih Nasseri <tahirih.nasseri@mail.utoronto.ca>
Subject: Authorship for AA Case Report Submission

Hi Team,

I received the following about our AA paper resubmission (I've attached the manuscript to the email for you to see the authorship order along with the table of changes). (see below in red).

> Please upload an MS Word or PDF file explaining the reason for the change and showing screenshots of each author's emailed consent to changing the authorship line, with the author(s) being added and the order of authors explicitly stated in each author's email. The author(s) who is being added must also consent to the change. The format of the email from each author could be as follows:
> "I [ADD Author First/Last Names] consent to [Adding] [Author First and Last Name] to the authorship line of JAAD manuscript, "[Add Manuscript Title]. I consent to the following author order: [Add all first/last names of authors in the correct order]"

We added Tahirih because she did a lot of editing for the manuscript revision and Sheila advised I add myself as third author as I made the figure, wrote and edited most of the manuscript.

I was wondering if you could send me back a statement with the following:
"I [**ADD Author First/Last Names**] consent to [Adding] [Tahirih Nasseri] to the authorship line of JAAD manuscript, "[AI-Based Alopecia Assessment: A Proof of Concept for Enhancing Accuracy and Objectivity in Hair Loss Measurement]. I consent to the following author order: [Evan Chan, BM1*, Kaitlyn Ramsay, PhD2*, Rachel Tyli, BASc3*, Ryan S.Q. Geng, MSc2, Tahirih Nasseri, BSc 2, Vincent Piguet MD, PhD, FRCP 5, Robert D.J. Fraser, BScN, MN, RN4,5, Sheila C. Wang, MD, PhD, FRCPC2,4,6,] *all authors contributed equally"

Sheila, if you could send this email to Vincent, that would be great. I'm trying to track everyone else down on my end. So far, Tahirih and Evan have sent me back their emails.

Best,
Rachel

CONFIDENTIALITY NOTICE: This email and any attachments are confidential and intended solely for the named recipient(s). If received in error, please notify the sender and delete the email from your system. This email may contain protected health information (PHI) under HIPAA, PHIPA, and applicable laws. Unauthorized use, reproduction, or distribution is strictly prohibited.

Subject: RE: Authorship for AA Case Report Submission
Date: Sunday, August 3, 2025 at 11:53:54 Eastern Daylight Saving Time
From: Vincent Piguet
To: Sheila Wang, Rachel Tyli

Fine by me. Vincent

From: Sheila Wang <sheila.wang@utoronto.ca>
Sent: July 30, 2025 6:56 PM
To: Vincent Piguet <vincent.piguet@utoronto.ca>; Rachel Tyli <rachel.tyli@utoronto.ca>
Subject: Fwd: Authorship for AA Case Report Submission

Hi Vincent
I have added a medical student, Tahirih Nasseri, to the authorship list for her contributions to our manuscript revisions - if you're ok with the authorship change please would you send Rachel (cc'd here) the following statement from you:

> "I [**ADD Author First/Last Names**] consent to [Adding] [Tahirih Nasseri] to the authorship line of JAAD manuscript, "[AI-Based Alopecia Assessment: A Proof of Concept for Enhancing Accuracy and Objectivity in Hair Loss Measurement]. I consent to the following author order: [Evan Chan, BM1*, Kaitlyn Ramsay, PhD2*, Rachel Tyli, BASc3*, Ryan S.Q. Geng, MSc2, Tahirih Nasseri, BSc 2, Vincent Piguet MD, PhD, FRCP 5, Robert D.J. Fraser, BScN, MN, RN4,5, Sheila C. Wang, MD, PhD, FRCPC2,4,6,] *all authors contributed equally"

> Please upload an MS Word or PDF file explaining the reason for the change and showing screenshots of each author's emailed consent to changing the authorship line, with the author(s) being added and the order of authors explicitly stated in each author's email. The author(s) who is being added must also consent to the change. The format of the email from each author could be as follows:
> "I [ADD Author First/Last Names] consent to [Adding] [Author First and Last Name] to the authorship line of JAAD manuscript, "[Add Manuscript Title]. I consent to the following author order: [Add all first/last names of authors in the correct order]"

Thank you

Sheila

Subject: Re: Authorship for AA Case Report Submission
Date: Wednesday, July 30, 2025 at 18:42:38 Eastern Daylight Saving Time
From: Sheila Wang
To: Rachel Tyli
CC: Rob Fraser, Wang QH, Ryan Geng, Tahirih Nasseri

"I Sheila Wang consent to adding Tahirih Nasseri to the authorship line of JAAD manuscript, "[AI-Based Alopecia Assessment: A Proof of Concept for Enhancing Accuracy and Objectivity in Hair Loss Measurement]. I consent to the following author order: [Evan Chan, BM1*, Kaitlyn Ramsay, PhD2*, Rachel Tyli, BASc3*, Ryan S.Q. Geng, MSc2, Tahirih Nasseri, BSc 2, Vincent Piguet MD, PhD, FRCP 5, Robert D.J. Fraser, BScN, MN, RN4,5, Sheila C. Wang, MD, PhD, FRCPC2,4,6,] *all authors contributed equally"

Sheila Wang, MD PhD FRCPC
Assistant Professor & Clinician Investigator, University of Toronto
Dermatologist, Women's College Hospital
Senior Fellow, Massey College
Co-Founder, Swift Medical

> On Jul 30, 2025, at 6:30PM, Rachel Tyli <rachel.tyli@utoronto.ca> wrote:
>
> "I [**ADD Author First/Last Names**] consent to [Adding] [Tahirih Nasseri] to the authorship line of JAAD manuscript, "[AI-Based Alopecia Assessment: A Proof of Concept for Enhancing Accuracy and Objectivity in Hair Loss Measurement]. I consent to the following author order: [Evan Chan, BM1*, Kaitlyn Ramsay, PhD2*, Rachel Tyli, BASc3*, Ryan S.Q. Geng, MSc2, Tahirih Nasseri, BSc 2, Vincent Piguet MD, PhD, FRCP 5, Robert D.J. Fraser, BScN, MN, RN4,5, Sheila C. Wang, MD, PhD, FRCPC2,4,6,] *all authors contributed equally"
